# Arthroscopic Hip Capsule Closure Augmented With a Nonwoven Polyethylene Terephthalate Scaffolding

**DOI:** 10.1002/atn2.70254

**Published:** 2026-09-11

**Authors:** Sean McMillan, Elizabeth Ford

**Affiliations:** ^1^ Virtua Health Marlton New Jersey U.S.A.; ^2^ Allegheny Health Network Pittsburgh Pennsylvania U.S.A.

## Abstract

The importance of hip capsule closure after arthroscopy has increasingly been shown to improve patient‐reported outcomes and decrease the risk of revision surgery. Nevertheless, not all surgeons close the capsule, and not all closures heal appropriately. Reasons for lack of closure and or closure failure include deficient tissue quality and the procedure being technically demanding. The purpose of this technical note is to show an innovative capsule closure during hip arthroscopy via the use of a nonwoven polyethylene terephthalate augment that is secured to the native capsular tissue using a “felting needle” mechanism. Our technique describes a user‐friendly method of providing a mechanically strong augmentation to the repair at time zero.

## Graphical abstract

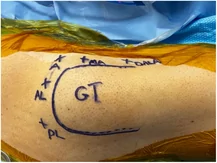

**VIDEO 1:** The case is a 38‐year‐old competitive horseback rider with Ehlers‐Danlos syndrome who previously underwent left hip labral repair with capsule closure 4 years prior. After sustaining a traumatic reinjury 8 months ago, magnetic resonance imaging arthrogram showed incompetency of the hip capsule, and decision was made to revise the capsule closure and place an augment to reinforce the repair. With the hip under 75 pounds of traction on a postless table, a 70° arthroscope is inserted into the anterolateral portal and survey is taken of the previous repair and the joint. A capsule blade is introduced through the midanterior portal, and the previous capsular repair is reopened connecting the portals and the insufficient tissue. Working through the midanterior portal, a propriety hip capsule suture passing device is used to place nonabsorbable #2 sutures sequentially through the near and far side of the capsule. Once the desired number of sutures are placed, the traction is taken down, and the suture limbs are tied from anterior to posterior. Next, the distal anterolateral accessory portal is created, and a cannula is placed. Through the 8‐mm cannula, a nonwoven polyethylene terephthalate augment measuring 23 × 30 mm in width and length respectively is introduced using a back‐grasping device. The augment is placed over the repair site, and a proprietary felting needle gun is inserted through the midanterior portal. Gentle pressure is placed on the augment with the gun as the firing mechanism is activated and the needle drives fibers of the polyethylene terephthalate augment into the patient's native capsule tissue, securing the augment with hundreds of points of contact. The gun is ideally used 45° to 90° to the augment to maximize fiber penetration into the capsule. Once fixation is complete, the hip is taken through a full range of motion to evaluate stability. Video content can be viewed at https://doi.org/10.1002/atn2.70254.

Advances in hip arthroscopy have allowed for minimally invasive treatment of various osseous and soft tissue pathologies. Contouring of bony abnormalities associated with femoral acetabular impingement and repair of damaged labral tissue has garnered most of the focus among hip arthroscopists. However, as indications have expanded, attention has shifted toward the role of the hip capsule in maintaining joint stability and normal biomechanics.

Capsulotomy is routinely performed during hip arthroscopy to facilitate visualization and instrument maneuverability. However, disruption of the ligamentous complex that makes up the hip capsule can alter native hip biomechanics. Two commonly methods of performing capsulotomy are either transverse connection of the arthroscopic portals or a “T” capsulotomy which can allow for further exposure of the femoral neck. Failure to close the capsule, particularly “T” capsulotomies, has been implicated as a potential contributor to postoperative instability, microinstability, and persistent pain.^1^, ^2^ Biomechanical studies have showed increased femoral head translation and rotational laxity after capsulotomy, particularly when the iliofemoral ligament is violated, raising concern regarding the long‐term consequences of unrepaired capsular defects.^3^, ^4^

In response, capsular repair and closure techniques have gained increasing attention. Cadaveric and in vivo biomechanical investigations suggest that capsular closure can partially or fully restore hip stability after arthroscopic capsulotomy.^5^ Clinically, emerging evidence has associated capsular repair with improved patient‐reported outcomes (PROMs), particularly in young, active patients and those with underlying ligamentous laxity or borderline acetabular dysplasia.^6^, ^7^, ^8^ Despite these findings, capsular management remains variable among hip arthroscopists and is often perceived as more technically demanding than labral repair. Failure of hip capsule repair has been associated with decreased patient outcomes and attributed to poor tissue quality, surgical technique, and patient factors.^2^, ^9^

The purpose of this technical note is to describe a reproducible method of capsular management and repair after addressing intra‐articular pathology. The authors describe a method of augmenting primary capsular repair with a nonwoven polyethylene terephthalate (PET) augment for reinforcement of the soft tissue repair.

## SURGICAL TECHNIQUE

### Positioning

The patient is positioned supine on a traction table using a high‐friction pad in a postless configuration (Guardian, Stryker Orthopedics) with 10° to 15° of Trendelenburg positioning. The feet are placed into well padded “ski‐boots” and secured to the traction extenders. The operative limb is maintained in neutral adduction and 10° of internal rotation, while the nonoperative limb is positioned in approximately 20° of abduction. Care is taken to ensure the C‐arm can maneuver over the patient's pelvis without metal artifact interference. The ALP is established just anterior to the greater trochanter under fluoroscopic guidance, followed by creation of the midanterior portal (MAP) and distal anterolateral accessory portal (DALAP) under direct arthroscopic visualization with fluoroscopic assistance (Figure 1).

**FIGURE 1:**
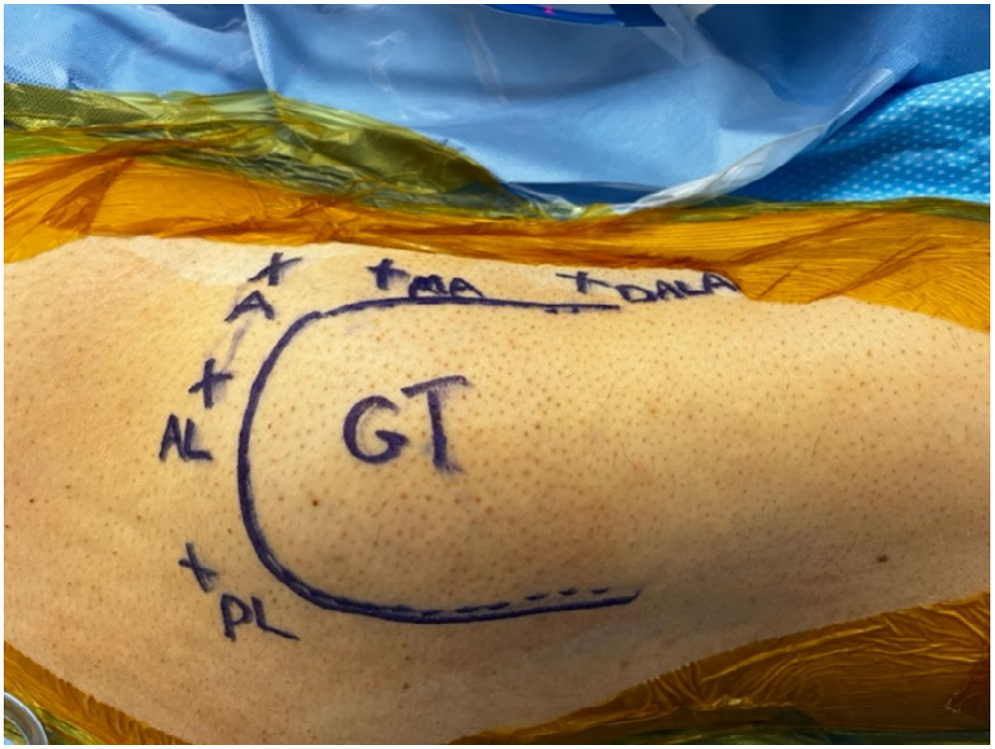
A right hip (patient head to left, feet to right of image) has been marked out with traditional portals. Hip access for viewing is carried out through the ALP, with working portals created through the MA and DALAP. (ALP, anterolateral portal; DALAP, distal anterolateral accessory portals; GT, greater trochanter; MA, midanterior.)

### Capsular Preparation

Transverse capsulotomy is created to facilitate introduction of instruments into the hip joint to address the osseous and labral abnormalities (Figure 2). After completion of this, attention is then turned to repairing the hip capsule (Video 1). The 70° arthroscope, viewing through the ALP, is withdrawn into the extracapsular space. Cannulas are maintained in the MAP and the DALAP to facilitate the capsule repair.

**FIGURE 2:**
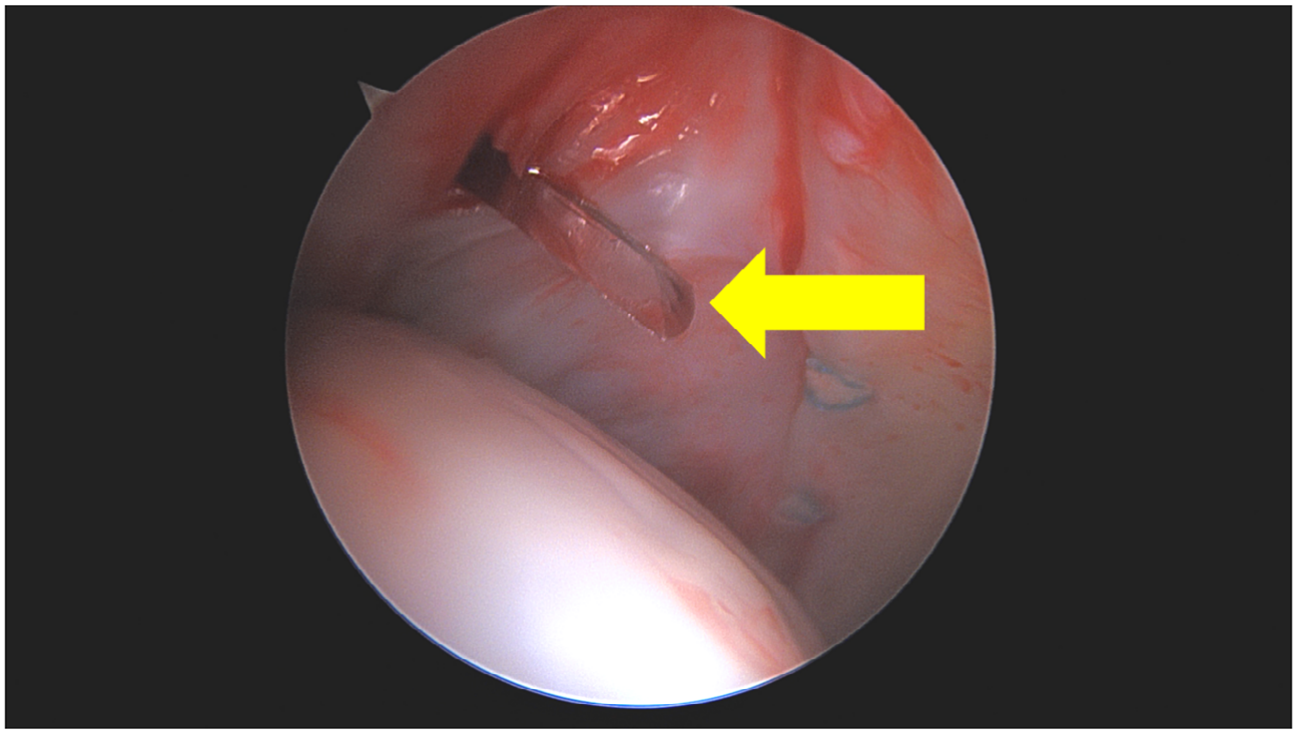
Viewing through the ALP on a left hip in the supine position, a capsular blade (yellow arrow) is introduced through the MAP to connect these portals to facilitate instrument introduction to the joint. This patient had undergone a previous labral repair and capsule closure which had a traumatic disruption postoperatively. (ALP, anterolateral portal; MAP, midanterior portal.)

Working through the MAP cannula with the hip under traction, a proprietary suture passing device (SlingShot, Stryker, San Jose, CA) is delivered through the far side (away from the acetabulum) capsular tissue. After passing the first #2 suture, the device is then passed top‐down through the near‐side (acetabular side) capsular tissue and the suture is retrieved completing the first repair sequence. Additional sutures are placed in a similar manner working from anterior to posterior (Figure 3). Once the desired number of sutures has been placed, traction is released and the stitches are tied sequentially using standard arthroscopic knot tying techniques.

**FIGURE 3:**
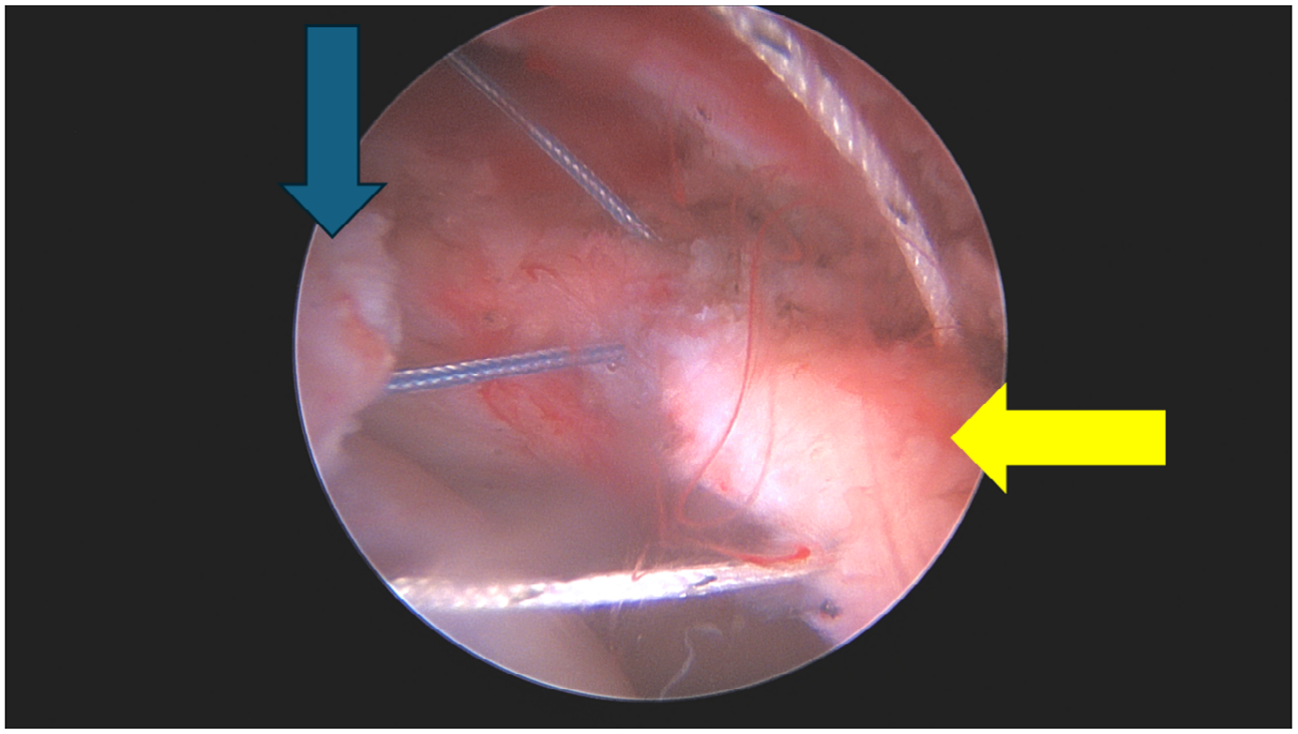
Viewing through the ALP on a left hip in the supine position, sequential #2 sutures have been passed through the near (yellow arrow) and far side (blue arrow) of the capsule for repair. (ALP, anterolateral portal.)

### Augmentation

After completion of the primary capsular repair, the 30 × 23 mm PET scaffold (SpeedPatch, ZuriMED Technologies AG) is introduced through the DALAP using an arthroscopic grasper. Once positioned over the repair site, a propriety “felting” needle gun (FiberLocker, ZuriMED Technologies AG) is introduced through the MAP (Figure 4). The gun and felting needle are passed with pressure over the scaffolding for a period of 90 seconds, driving fibers of the scaffolding into the capsule 7 to 8 mm deep. The gun can be maneuvered between portals to ensure it contacts the scaffold at an angle of 45° to 90° to ensure maximal fiber integration of the tissue. After completion, the hip is then taken through a full range of position to ensure repair stability (Figure 5).

**FIGURE 4:**
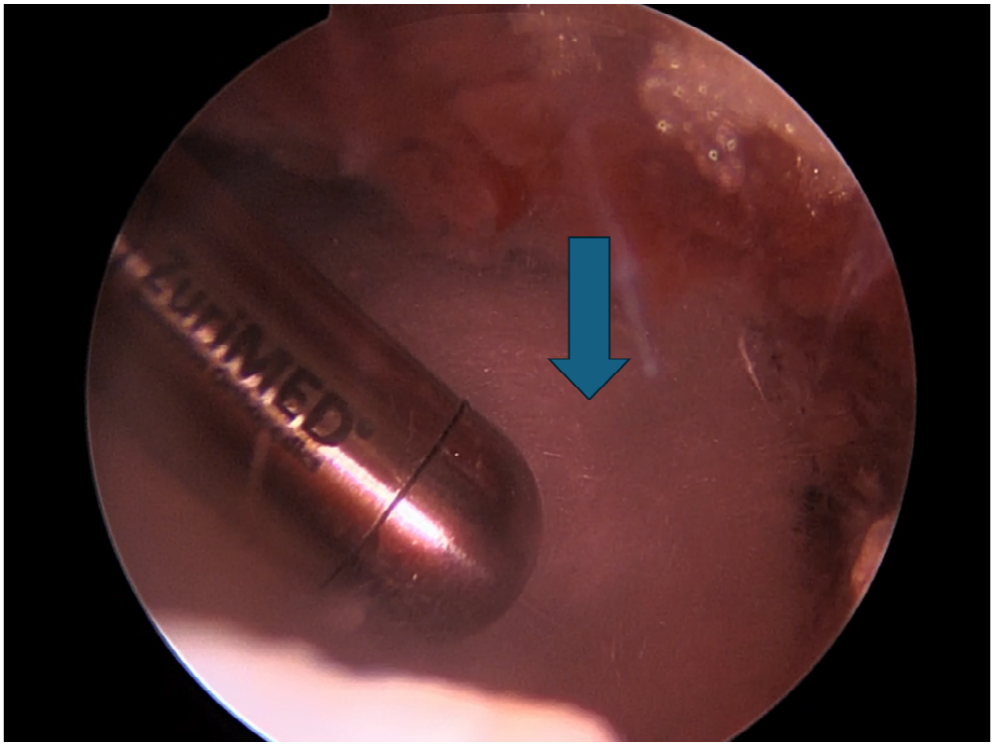
Viewing through the ALP on a left hip in the supine position, the patch augment (blue arrow) has been placed over the repair through the DALAP. The penetrating gun that will drive the fibers of the patch into the native capsule tissue is placed through the MAP. (ALP, anterolateral portal; DALAP, distal anterolateral accessory portals; MAP, midanterior portal.)

**FIGURE 5:**
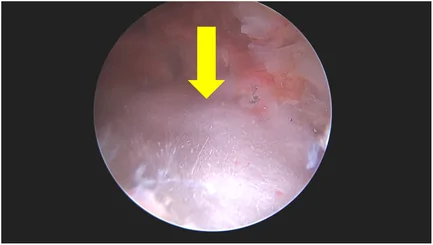
Viewing through the ALP on a left hip in the supine position, a final survey is performed noting the PET patch augment (yellow arrow) firmly secured to the native capsule tissue. (ALP, anterolateral portal; PET, polyethylene terephthalate.)

## DISCUSSION

Hip capsular closure is an important portion of the hip arthroscopy procedure. Numerous articles have reported improved PROMs as well as a decrease in revision rates. The integrity of the capsule is thought to play a role in reduction of microinstability of the hip joint. Specifically, biomechanical studies have showed the crucial role the iliofemoral ligament plays in hip stability, emphasizing the importance of capsular restoration.^3^, ^4^

Looney et al. performed the largest meta‐analysis to date, examining 5132 hip arthroscopic procedures, and noted hip capsular repair was associated with significantly higher post‐PROMs.^6^ Specifically, patients who had hip capsule closure showed higher modified Harris Hip Score, Hip Outcome Score‐Activities of Daily Living, and Hip Outcome Score‐Sports Specific Subscale compared with those without repair.

Economopoulos et al. conducted a prospective randomized controlled examining capsular repair versus unrepaired T‐capsulotomy.^7^ Their prospective trial identified superior modified Harris Hip Score (86.2 vs 76), Hip Outcome Score‐Activities of Daily Living (85.6 vs 76.8), and Hip Outcome Score‐Sports Specific Subscale (74.4 vs 65.3) at 2‐year follow‐up compared with the repair group. Furthermore, they found that 4 of the unrepaired patients were eventually converted to total hip arthroplasty compared with 0 patients in the repair cohort. Confirming these findings, multiple systematic reviews have showed that hip capsule closure is associated with improved survivorship and reduced revision rates.^8^, ^10^, ^11^, ^12^, ^13^ Domb et al. reported in their systematic review greater overall improvements in PROMs and higher survivorship rates (94.6%‐100% vs 90.8%‐100%) compared with those without repair.^5^ Boos et al. significantly reported total hip conversion rates of 31% compared with 3% in those who did not have their capsule repaired.^11^ In high‐risk populations, such as high‐level athletes, Hassbrook et al. reported faster return‐to‐play rates (4.7 vs 5.8 months) and higher return‐to‐play rates (90.3% vs 75.5%) in patients who underwent closure compared with those who did not.^13^

Despite the mounting evidence to suggest the benefit of hip capsule repair, several factors have been associated with hip capsular repair failure that can lead to challenges for the treating surgeon. Some of the most common reasons for capsular repair failure are tissue deficiency and the technically challenging nature of the repair. Reasons for deficiency include poor capsulotomy creation, revision cases, capsulolabral adhesions, traumatic injury, and residual bony or labral pathology.^2^, ^9^

To aid in repair of the hip capsule, the authors have described a technique that is designed to augment traditional suture repair of the capsule. The purpose of the augmentation is to maximize the initial closure strength and provide a reproducible method of support for deficient tissue or difficult closures. The system uses a nonwoven PET patch (SpeedPatch) and a specialized FiberLocker Instrument. The instrument pushes individual patch fibers into the underlying tendon, creating strong fixation without traditional sutures for the patch itself, increasing immediate repair strength (Figures 6 and 7). The composition of the patch allows for it to be placed and maintain its position without suture fixation if desired (Table 1).

**FIGURE 6:**
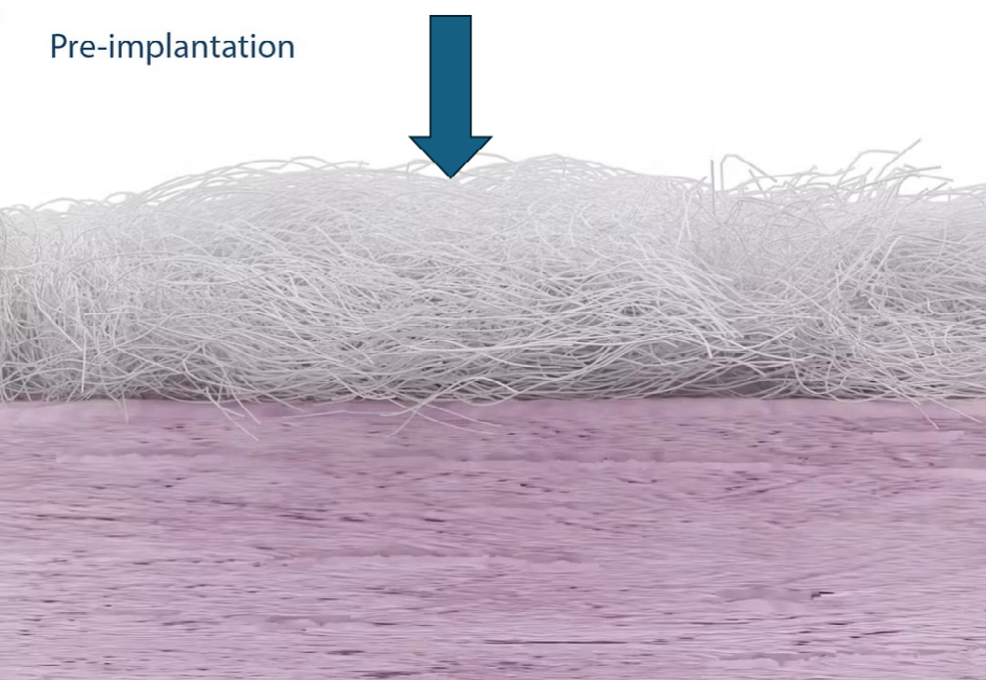
Pictured is the nonwoven PET augment (blue arrow) on top of a simulated tendon. The augment is made up of hundreds of the PET fibers that can be delivered into the tendon using a propriety felting needle gun (image reprinted with permission from ZuriMED Technologies AG). (PET, polyethylene terephthalate.)

**FIGURE 7:**
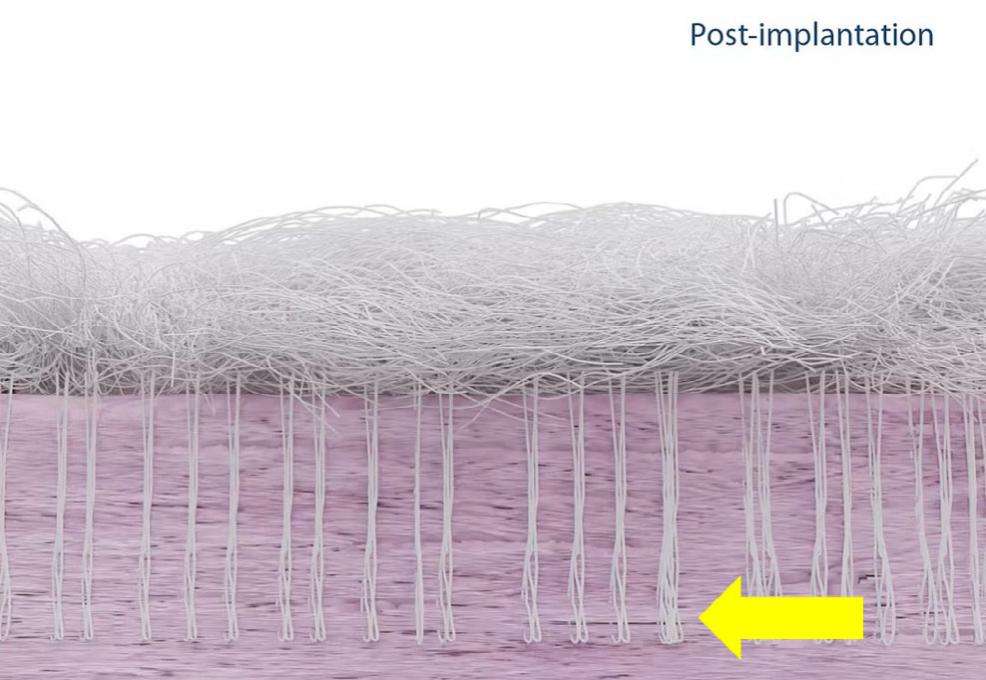
Via the use of a propriety felting needle gun, fibers of the PET augment have been driven into the tendon approximately 8 mm in depth (yellow arrow). The end product of this mechanism of augment to tissue fixation is hundreds of points of contact, thereby increasing mechanical strength of the repair at time zero (image reprinted with permission from ZuriMED Technologies AG). (PET, polyethylene terephthalate.)

**TABLE 1:** Advantages and Disadvantages of This Surgical Repair Augmentation of the Hip Capsule Using a PET Scaffolding Are Addressed Below

Advantages	Disadvantages
The implant shows tremendous ease of delivery and fixation	The implant does not resorb or degrade
The implant offers time zero strength to the repair	The implant is not a biologic augment
Fixation of the implant requires only 90 seconds to obtain 3600 points of fixation to the native tissue	PET implants have yet to be studied biomechanically in terms of its compatibility to the native hip capsule
Implant can be placed without the need for sutures or anchors	Currently, the fixation gun comes in only 1 length. In patients with large body habitus, a longer working length may be required

PET, polyethylene terephthalate.

The authors acknowledge limitations with this technique. Although the implant shows promise, to date, there have been studies examining its use in the hip (Table 2). Further studies are warranted to examine the biomechanical strength of the augment in conjunction with the capsule. Additionally, prospective studies looking at the potential benefit and risk associated with the augment on PROMs should be performed before considering widespread adoption. Nevertheless, the superior strength the implant has shown in the rotator cuff model, combined with its ease of use, provides potential benefit in the right clinical setting for physicians as they address the hip capsule after arthroscopy.

**TABLE 2:** “Pearls and Pitfalls” of Surgical Repair Augmentation of the Hip Capsule Using a PET Scaffolding Are Addressed Below

Pearls	Pitfalls
Clear the periarticular space to allow for clear visualization and avoid possible impediments to deliver/implantation	The implant is secured to the tissue through the needle within the delivery gun. It penetrates a depth of 8 mm and works best in a perpendicular plane to the implant. A flat trajectory will cause skiving and lead to potential failure
Use a spinal need to ensure the appropriate trajectory to implant the scaffolding through the DALA portal	Implantation in patients with a large body habitus may prove challenging as the fixation gun has only 1 working length, designed for shoulder
Consider flexing the hip to 15°‐30° during the initial capsular closure to take tension of the repair and also avoid overtightening the capsule when the augment is affixed to the native tissue	Attempting to “blend” the SpeedPatch implant with another biologic patch can lead to failure of the construct if the second patch is too thick because of the delivery gun only being able to penetrate a depth of 8 mm

DALA, distal anterolateral accessory portals; PET, polyethylene terephthalate.

## DISCLOSURES

The authors (S.M., E.F.) declare that they have no known competing financial interests or personal relationships that could have appeared to influence the work reported in this article.

## References

[1] Owens JS , Jimenez AE , Shapira J , et al. Capsular repair may improve outcomes in patients undergoing hip arthroscopy for femoroacetabular impingement: A systematic review of comparative outcome studies. Arthroscopy. 2021;37:2975‐2990. 10.1016/j.arthro.2021.03.063 33887416

[2] Malempati M , Terle PM , Dhillon J , Kraeutler MJ . Residual structural disease and new labral tears are the most common indications for revision hip arthroscopy: A systematic review. Arthroscopy. 2025;41:5437‐5452.e2. 10.1016/j.arthro.2025.07.024 40738255

[3] Beck EC , Suppauksorn S , Nho SJ . The role of comprehensive capsular management in hip arthroscopy for the treatment of femoroacetabular impingement syndrome. Arthroscopy. 2020;36:9‐11. 10.1016/j.arthro.2019.10.028 31864606

[4] Riff AJ , Kunze KN , Movassaghi K , et al. Systematic review of hip arthroscopy for femoroacetabular impingement: The importance of labral repair and capsular closure. Arthroscopy. 2019;35:646‐656.e3. 10.1016/j.arthro.2018.09.005 30712639

[5] Carbone AD , Prabhavalkar O , Chishti Z , Curley AJ , Parsa A , Domb BG . Hip capsular repair results in improved patient‐reported outcomes and survivorship: A systematic review of the literature. Arthroscopy. 2023;39:488‐497. 10.1016/j.arthro.2022.11.013 36395962

[6] Looney AM , McCann JA , Connolly PT , Comfort SM , Curley AJ , Postma WF . Routine capsular closure with hip arthroscopic surgery results in superior outcomes: A systematic review and meta‐analysis. Am J Sports Med. 2022;50:2007‐2022. 10.1177/03635465211023508 34403279

[7] Economopoulos KJ , Chhabra A , Kweon C . Prospective randomized comparison of capsular management techniques during hip arthroscopy. Am J Sports Med. 2020;48:395‐402. 10.1177/0363546519894301 31891553

[8] Ortiz‐Declet V , Mu B , Chen AW , et al. Should the capsule be repaired or plicated after hip arthroscopy for labral tears associated with femoroacetabular impingement or instability? A systematic review. Arthroscopy. 2018;34:303‐318. 10.1016/j.arthro.2017.06.030 28866345

[9] Makhni EC , Ramkumar PN , Cvetanovich G , Nho SJ . Approach to the patient with failed hip arthroscopy for labral tears and femoroacetabular impingement. J Am Acad Orthop Surg. 2020;28:538‐545. 10.5435/JAAOS-D-16-00928 32574474

[10] Cohen D , Comeau‐Gauthier M , Khan A , et al. A higher proportion of patients may reach the MCID with capsular closure in patients undergoing arthroscopic surgery for femoroacetabular impingement: A systematic review and meta‐analysis. Knee Surg Sports Traumatol Arthrosc. 2022;30:2425‐2456. 10.1007/s00167-022-06877-9 35122108

[11] Boos AM , Wang AS , Lamba A , et al. Long‐term outcomes of primary hip arthroscopy: Multicenter analysis at minimum 10‐year follow‐up with attention to labral and capsular management. Am J Sports Med. 2024;52:1144‐1152. 10.1177/03635465241234937 38516883

[12] Kaplan DJ , Fenn TW , Jan K , Nho SJ . Capsular repair is associated with lower revision rates yet similar clinical outcomes and arthroplasty conversion 5 years after hip arthroscopy: A systematic review. Arthroscopy. 2023;39:1882‐1891.e1. 10.1016/j.arthro.2023.04.016 37146665

[13] Hassebrock JD , Makovicka JL , Chhabra A , et al. Hip arthroscopy in the high‐level athlete: Does capsular closure make a difference? Am J Sports Med. 2020;48:2465‐2470. 10.1177/0363546520936255 32667821

